# Paediatric Major Incident Planning at a Major Trauma Centre; Learning from Simulation

**DOI:** 10.15694/mep.2018.0000232.1

**Published:** 2018-10-15

**Authors:** Ruth Bird, Jo Lowlor, Breda O'Neill, Ami Parikh, Gin Peh, Naomi Edmonds

**Affiliations:** 1 Royal London Hospital

**Keywords:** Major incident, Trauma, Simulation, Paediatrics, PICU, Critical care, Anaesthetics, Major Trauma Centre

## Abstract

This article was migrated. The article was marked as recommended.

In light of the recent terrorist attacks in London and Manchester we wanted to test our Major Incident protocol for Paediatrics using simulation. A hospital wide MI Sim was completed in January but focusing on admission pathways, flow and theatre.

As a follow up exercise, we wanted to test discharge pathways and our immediate bed capacity in PICU and paediatrics, allowing us to, assess immediate capacity, estimate discharge numbers, test communication pathways, staff responces and next shift planning as well as identifying issues allowing us to improve our major incident policy.

## Introduction

A major incident is any event whose impact cannot be handled within routine service arrangements. It requires the implementation of special procedures by one or more of the emergency services, the NHS, or a Local Authority to respond to it.

These are events that are unpredictable, sudden, and that result in a large number of injured or ill casualties presenting to the emergency services over a short period of time.

Previously it has been shown by Carley that the standard of preparation for major incidents in the UK could be improved.This may have in part because of the fact that major incidents were perceived to be rare events and therefore preparation was of low priority.

However, with the increase in terrorist related incidents in the last few years, incidents have increased awareness in the importance of preparation. Planning and training have been repeatedly cited as essential components to a successful response to a major incident, particukarlly by Rowland et al who stressed the importance of “readiness”.

Major Incident Plans should be reviewed and updated (where necessary) at least annually, following exercises or after an incident has occurred. The Trusts approach to emergency preparedness should be assessed on a regular basis.

In light of the recent terrorist attacks in London and Manchester we wanted to test our Major incident (MI) protocol specifically for paediatrics using a simulated exercise (SIM). Manchester in particular highlighted that attacks could be targeted at children and young adults and that these types of incidents created unique problems.

A hospital wide MI SIM was completed in January 2018 but focused on admission pathways, flow and theatre utilisation. As a follow up exercise, we wanted to test discharge pathways and our immediate bed capacity in the paediatric intensive care unit (PICU) and the paediatrics wards.

The Paediatric specific Major Incident pathway was designed and circulated. It allocates on shift doctors and nurses not involved in resuscitation cases, to “area based” bed meetings at the initiation of a MI, allowing the identification of patients able to be discharged / step down within the first 30 minutes of the incident, allowing time for the completion of discharge paperwork and bed turnover.

### The AIMS of the exercise were to:-

• Assess immediate bed capacity
• Estimate discharge numbers for the Paediatric Intensive Care Unit (PICU) and the wards
• Test Communication pathways
• Identify problems / issues
• Test Staff responses to communication pathways
• Test awareness of next shift planning

## Method

We ran a live exercise testing the new MI policy using current in patient demographics from bed boards and handover sheets to assess discharge capacity in real time for bleep holders and nurses in charge.

The major incident involved a table-top based simulation involving 16 children <16yrs of age (the cut off for paediatric patients at the Royal London Hospital).

There were 2 ventilated patients requiring PICU, 3 needing immediate theatre, likely to require post-operative care on PICU, 4 awaiting theatre and 7 to be admitted straight to the wards. Discharge planning meetings began at the moment of the Major Incident simulation bleep. Nursing and medical staff used watts app to arrange next shift planning and recruit staff members to respond with potential to attend.

**Image 1.**
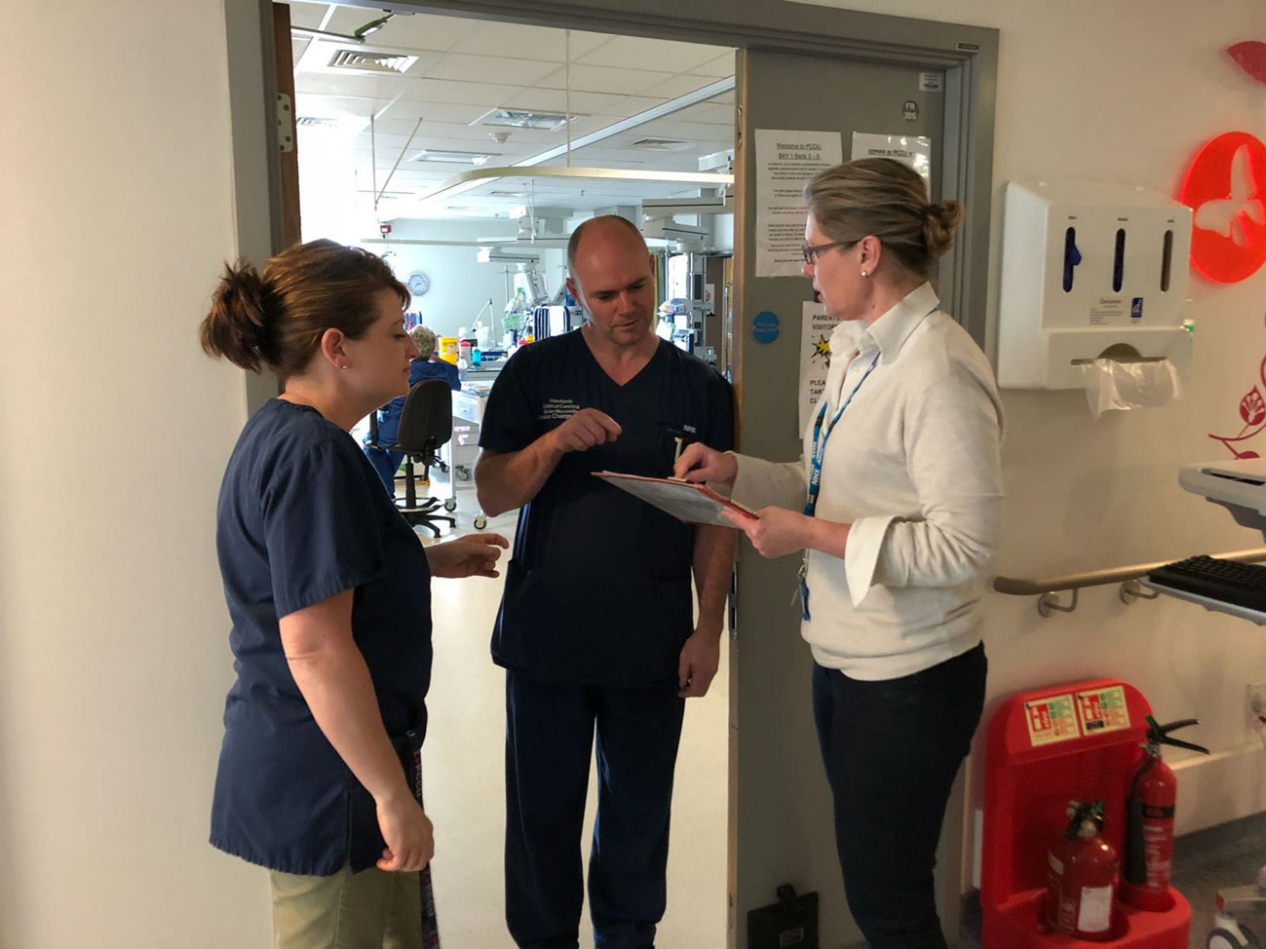
Major incident planning on PICU

Consent to use photograph was obtained from all staff members.

## Results/Conclusion

33 in-patients were identified as potentially dischargeable during the exercise. 26 of these were felt to be immediately dischargeable with to take away paperwork/prescriptions (TTA’s).

During the exercise the paediatric intensive care unit had seven out of their eight commissioned bed spaces occupied. They had a potential for 2 more physical bed spaces in the event of Major incident. They quickly identified 3 patients that were able to be stepped down.

Our communication pathway for nursing staff also had a good response identifying 33 nurses able to attend work within 1 hr of the call allowing adequate shift planning for an ongoing response post incident.

## Discussion

This capacity is likely adequate to accept paediatric patients in the event of a major incident assuming PICU has the required space for critically unwell children on the given day. Regardless of immediate capacity, the utilisation of the extra two bed spaces, should, be able to be covered by the extra nursing staff available within the first hour. A hot debrief allowed the team to discuss any difficulties face and make suggestions for future practise.

This simulation itself allowed us to identify the need for a rolling sheet to provide basic information about patient demographics, diagnosis, treatment and outstanding jobs for patient’s transfers to other wards.

We also identified the need for a quick paper copy TTA’s for use in Major Incident and have sourced printed carbon copy paper to facilitate quick transcription, with a copy for the patient, the medical notes, and a Major Incident box, allowing follow up post discharge and an electronic format TTA to be completed at a later date as these are timely to complete.

We liaised with lead pharmacists to get or emergency prescription pads for discharge medications meaning our in-patient pharmacy does not become overwhelmed in the event of a mass casualty event.

The updated protocol contains action cards for all staff including clinical specialists / therapies teams / doctors / nurses and admin staff.

The procedures and protocols outlined in the plan can be used to respond to an incident.

The command Hub communication pathways ran smoothly with nursing staff, doctors and bed management teams up to date with potential discharges, transfers and needs of the wards.

We would encourage trauma facilities to train for major incidents using simulation to identify pitfall and generate solutions to difficulties faced locally during these times. Paediatric patients create unique challenges in major incidents, particularly related to safe discharge planning.

## Take Home Messages

• We would encourage trauma facilities to train for major incidents using simulation to identify pitfall and generate solutions to difficulties faced locally during these times.

• Paediatric patients create unique challenges in major incidents, particularly related to safe discharge planning.

## Notes On Contributors

Ruth Bird is currently completing an Anaesthetics fellowship job in Paediatric Trauma. Her projects include updating the Paediatric Major Incident plan for the Royal London Hospital.

Jo Lowlor is currently Matron for Paediatrics on The Royal London Site. Jo has worked at The Royal London Hospital for 18 years and has been ward manager for 8yrs. She has an interest in improving patient flow through the Paediatric service in order to better patient and family experience.

Ami Parikh is a Paediatric ED consultant at the Royal London Hospital dedicated to improving patient care.

Gin Peh has been a Paedaitric Consultant at the Royal London Hospital since 2016, he trained at the university of dundee.

Breda O’Neill is a Paediatric and Adult Anaesthetist at the Royal London specialising in Trauma.

Naomi Edmonds is a duel trained PICU and Anaesthetics consultant with a special interest in trauma and major incident.

## References

[ref1] Carley SD Mackway-Jones K. (1996) Are we ready for the next major incident: Analysis of hospital major incident plans. BMJ. 313:1242- Available at https://www.ncbi.nlm.nih.gov/pmc/articles/PMC2352552/( Accessed: 3rd Oct 2018). 8939115 10.1136/bmj.313.7067.1242 PMC2352552

[ref2] Carley S Mackway-Jones K Donnan S. (1998) Major incidents in Britain over the past 28 years: the case for the centralised reporting of major incidents. J Epidemiol Community Health. 52:392–398. 10.1136/jech.52.6.392 9764261 PMC1756719

[ref3] Lowe D Millar J Dignon N. Ireland A. (2016) Top 10 lessons from the Glasgow major incidents. Emerg Med J. 33(8)596–597. 10.1136/emermed-2015-205626 26976660

[ref4] Nancekievill DG. (1992) On site medical services at major incidents. BMJ. 1992;305:726. 10.1136/bmj.305.6860.1015-a 1422324 PMC1883390

[ref5] Rowlands BJ. (1990) Are we ready for the next disaster. Injury. 21:61–2. 10.1016/0020-1383(90)90158-Q 2347637

[ref6] Rutherford WH. (1990) The place of exercises in disaster management. Injury. 21:58–60. Available at https://www.ncbi.nlm.nih.gov/pubmed/2347636( Accessed: 3rd Oct 2018). 2347636 10.1016/0020-1383(90)90157-p

