# Supplementary material for: Paediatric Major Incident Planning at a Major Trauma Centre; Learning from Simulation
Source: MedEdPublish (2016). 2018 Oct 15;7:232. [Version 1] doi: 10.15694/mep.2018.0000232.1 (PMC10711939; doi:10.15694/mep.2018.0000232.1)
Supplement: Peer review history

# Reviewer report: Reviewer response for version 1

**DOI:** 10.21956/mep.19687.r29419

**Published:** 2018-10-31

**Authors:** Juan Martin

**Affiliations:** 1 Newham University Hospital

This review has been migrated. The reviewer awarded 5 stars out of 5 This is a good example of medical training that every hospital should put in practice.Taking into consideration the limited space for publication, it is a very well structured article and, I think the authors should be allowed to upload some documents / protocols that they used for the SIM: structure of the teams, distribution of duties, network with emergency services, etc, that may benefit other hospitals.I am sure that in the "hot debrief" there were a lot of proposals for improvement, but the main question should be: could we do it even better? Once these major events can not be prevented, what would be the maximum capacity (limited a lot of times by the number of ventilators)?. I am just thinking that Royal London Hospital belongs to a Trust / Network of hospitals that may have capacity to take those 7 patients from the ward and 3 stepped down from PICU. This possibility should be explored because 10 more spaces in a specialised center like Royal London could be of great value.

Reviewer Expertise:

NA

No decision status is available

# Reviewer report: Reviewer response for version 1

**DOI:** 10.21956/mep.19687.r29418

**Published:** 2018-10-24

**Authors:** Stuart Marshall

**Affiliations:** 1 Monash University

This review has been migrated. The reviewer awarded 5 stars out of 5 Dr Bird and colleagues share an example of an important and often under-recognised purpose of simulation; that of process design and testing.Their case study describes how they tested their protocols and procedures for discharge planning and patient flow during major incidents. In doing so, they determined areas for improvement for the rapid discharge of patients and communication between members of staff when transferring patients.The approach is similar in many ways to how cognitive systems engineers have redesigned processes in other domains such as nuclear power generation and military settings. These have typically looked at information flow and examination of the process and its components as a ‘cognitive system’. Importantly, the paper asks not only ‘what can we do differently?’ but also ‘what did we do well?’. In other words, moving from a ‘Safety 1’ to a ‘Safety 2’ paradigm of practise redesign, changing the items that need changing and reinforcing and codifying the areas that are highly functioning. Evidence of resilient strategies within the environment such as identifying where additional bed spaces can be safety added, in a description similar to Wears and colleagues’ work in emergency department planning (https://www.researchgate.net/publication/297371141_Resilience_in_the_emergency_department). Tellingly, all members of the team were invited to contribute during the debriefing and in fact the ‘team’ was extended to include pharmacy as a key contributor.The authors should be congratulated for their work and I hope descriptions of initiative such as this become more frequent as the benefits of simulation beyond the purpose of education are more fully recognised.

Reviewer Expertise:

NA

No decision status is available
